# Evaluation of Human-Approved Active Pharmaceutical Ingredients Against Lactate Dehydrogenase from *Theileria annulata*

**DOI:** 10.1007/s11686-026-01326-w

**Published:** 2026-07-10

**Authors:** Selcan Akar, Maria Orlenco, Erennur Ugurel, Özkan Danış, Dilek Turgut-Balik

**Affiliations:** 1 Department of Bioengineering, Faculty of Chemical and Metallurgical Engineering, Yildiz Technical University, Davutpasa Campus, 34210 Istanbul, Türkiye; 2 Faculty of Engineering and Natural Sciences, Istanbul Atlas University, 34408 Istanbul, Türkiye; 3 Department of Biomedical Engineering, Faculty of Engineering and Natural Sciences, Istanbul Atlas University, 34408 Istanbul, Türkiye; 4 Department of Chemistry, Faculty of Sciences, Marmara University, 34722 Istanbul, Türkiye

**Keywords:** *Theileria annulata*, Theileriosis, Lactate dehydrogenase, Drug repurposing, One health, FDA-approved drugs

## Abstract

**Background**

Theileriosis, a tick-borne disease caused by *Theileria annulata*, leads to global economic losses in livestock, emphasizing an urgent need for the identification of new therapeutic agents with alternative mechanisms of action due to rising buparvaquone resistance combined with climate-driven spread. *T. annulata* lactate dehydrogenase (*Ta*LDH) has been selected as a promising target in the current study. As approximately half of the FDA-approved veterinary drugs are shared with human medicine, integrating drug repurposing has gained importance as an emerging strategy for the management of tick-borne diseases.

**Methods**

In this study, *in vitro* and *in silico* approaches are integrated to screen 21 Active Pharmaceutical Ingredients (APIs) from FDA-approved drugs against *Ta*LDH in the context of drug repurposing.

**Results**

Six of 21 the APIs showed ≥ 60% inhibition against *Ta*LDH (over 95% purity), particularly the proton pump inhibitor omeprazole, which exhibited both the highest inhibition percentage (73.36%) and the lowest binding energy (− 6.36 kcal/mol), with a consistency between experimental and computational results. To our knowledge, this study is the first to evaluate FDA-approved human therapeutics as potential *Ta*LDH inhibitors.

**Conclusion**

This preliminary *in vitro* enzymatic inhibition study suggests omeprazole as a potential therapeutic agent for theileriosis. However, comprehensive pharmacokinetic and pharmacodynamic analyses will further validate and advance these promising findings. Furthermore, APIs with ≥ 60% inhibition of *Ta*LDH may serve as promising leads for the future development of more potent anti-theilerial agents through targeted derivatization and lead optimization. In alignment with the One Health framework, evaluating human-approved active pharmaceutical ingredients as veterinary therapeutic against parasitic diseases may offer a sustainable, time and cost-efficient strategy.

## Introduction

Livestock-derived products account for around 50% of global agricultural production by value, meeting various user requirements, including animal-based foods, materials for daily use, and agricultural inputs [1]. Moreover, the increase in urban population is expected to drive changes in dietary habits, promoting a transition towards the consumption of nutrient-rich foods, particularly those sourced from livestock [2]. It is estimated that demand for livestock products, including milk, meat, and eggs, will account for up to 80% of global food consumption and production by 2050 [3]. Despite existing and anticipated demand, the industry faces the devastating impacts of climate change, one of the most critical environmental challenges of the century [4, 5]. One of the significant impacts of climate change on livestock farming is its direct contribution to the spread and resurgence of vector-borne diseases [1]. The adverse effects of vector-borne diseases on animal health are evident across a wide geographical area, causing economic losses in the livestock sector and restricting global socioeconomic progress [6, 7]. Among vector-borne diseases transmitted by hematophagous arthropods such as mosquitoes, ticks, and sandflies, animals are highly vulnerable to tick-borne diseases [5, 6]. Especially, tick-borne hemoparasitic diseases pose a threat to cattle welfare and productivity, constituting a serious problem for the sustainability of the global animal husbandry sector [8]. Tropical theileriosis, a tick-borne hemoparasitic disease caused by the protozoan parasite *Theileria annulata,* is associated with the most lethal outcomes and poses a severe risk to the cattle industry in tropical and subtropical areas [9, 10].

The pathogenesis of theileriosis involves complex stages initiated with the transmission of the etiological agent by *Hyalomma* ticks and culminating in the uncontrolled, continuous proliferation of transformed cells [11]. It is estimated that nearly 250 million cattle are at risk of tropical theileriosis, a disease that not only leads to mortality but also significantly reduces productivity and tractive force in affected animals [12–14]. Current approaches to combat tropical theileriosis include controlling tick vectors, vaccinating cattle, and therapeutic intervention with the hydroxynaphthoquinone antiprotozoal drug [15]. Although the implementation of control strategies and the long-term efficacy of immunization are debatable, drug administration dating back to the 1980s remains a prevalent method for treating tropical theileriosis [15, 16]. Among the medications used to treat tropical theileriosis, buparvaquone (BPQ) remains the most effective therapeutic agent [17, 18]. Despite the effectiveness, resistance to BPQ has been documented in Tunisia [19], Iran [20], Sudan [21], Egypt [22], Pakistan [23], Türkiye [15], and China [24]. The emergence of resistance across various geographical areas limits treatment options and renders the problem irresolvable. In this regard, there is an increasing demand for drugs targeting *T. annulata*, particularly those with mechanisms of action distinct from BPQ [18].

In the context of drug discovery against parasites, research focuses on identifying and targeting pathogen-specific metabolic pathways or enzymes that differ from host counterparts [25]. *Theileria* species belong to a group of protozoan parasites that strictly depend on anaerobic metabolism to accomplish the energy requirements, so the enzymes of the glycolysis pathway have the potential to be effective drug targets [25]. Lactate dehydrogenase (LDH), a key glycolytic enzyme that catalyzes the reduction of pyruvate to lactate under anaerobic conditions, exhibits structural differences between the parasite and its host [26–28]. The structural differences from the host, along with the essential role in parasite survival, render LDH an attractive target for drug development [28]. Moreover, LDH has been investigated as a pharmacological target against apicomplexans, including *Plasmodium falciparum* [29], *Toxoplasma gondii* [30], and *Babesia divergens* [31].

New drug development processes are time-consuming and require substantial investments [32]. The approach of drug repurposing (drug repositioning) outside their primary indication offers an opportunity to overcome challenges in drug development [33]. Shared biological features between humans and animals enable the repurposing of drugs across species for the treatment of cross-species infections [34]. Scott et al*.* report the overlap in pharmacological approaches between human and veterinary medicine, as approximately fifty percent of FDA-approved veterinary drugs are also authorized for use in humans [34]. Recently, the integration of drug repurposing and the One Health approach, which addresses human, animal, and environmental health as a whole, has been discussed in the context of veterinary medicine [35, 36]. Moreover, the One Health concept is crucial for the management of tick-borne diseases [7, 35].

Studies in the drug repurposing scope are frequently carried out in the context of potential pharmaceutical ingredients’ analysis through computational studies, *in vitro* evaluation of pharmaceutical ingredients’ activities, or integration of both approaches [33]. In this study, 21 Active Pharmaceutical Ingredients from FDA-approved drugs, hereafter referred to as APIs, with different indications in humans were selected, including compounds previously investigated for antibacterial or antiparasitic activities towards drug repurposing study. These 21 APIs were screened in vitro to assess their potential inhibitory effects against *Ta*LDH for the first time. APIs exhibiting over 60% inhibition were also analyzed *in silico* to evaluate their binding affinity to *Ta*LDH.

## Materials and Methods

### APIs Used in the *Ta*LDH Inhibition Study

The selection of the 21 FDA-approved active pharmaceutical ingredients (APIs) was based on their reported biological activities in the literature, including compounds previously investigated for either antibacterial or antiparasitic effects, as well as their structural and functional diversity across different therapeutic classes. These criteria were considered to ensure a broad chemical space for the initial screening of potential *Ta*LDH inhibitory activity. Accordingly, a total of 21 FDA-approved APIs were subjected to *in vitro* inhibition assays to evaluate their potential inhibitory effects against *Ta*LDH. The specific compound ID (CID) or substance ID (SID) serving as a unique identifier of 21 APIs listed in Table 1 was obtained from the PubChem database (Kim et al. 2025) and were procured from a commercial supplier.

**Table 1:** Properties of the 21 active pharmaceutical ingredients of FDA-approved drugs

Active pharmaceutical ingredients	Compound/substance ID	Pharmacological class
Lanzoprozol pellets (Chemo Laboratorios Liconsa, S.A., Spain)	CID: 3883	Proton pump inhibitor (stomach)
Albendazole (SeQuent Scientific Ltd., India)	CID: 2082	Anthelmintic
Praziquantel (Shanghai Jiayi Pharmaceutical Co., Ltd., China)	CID: 4891	Anthelmintic
Montelukast sodium (Unimark Remedies Ltd., Vapi, India)	CID: 23,663,996	Respiratory system drug
Carvedilol (Moehs Catalana, S.L., Spain)	CID: 2585	Cardiovascular system
Amitriptyline (Teva Api, Israel)	CID: 2160	Antidepressant
Amlodipine Besylate (Prudence Pharma Chem, India)	CID: 60,496	Cardiovascular system
Pantoprazole sodium (SMS Pharmaceuticals Ltd., India)	CID: 11,954,257	Proton pump inhibitor (stomach)
Nateglinide (Cadila Pharmaceuticals Ltd., India)	CID: 5,311,309	Type 2 diabetes
Buspirone hydrochloride (Alkaloida Chemical Company Zrt., Hungary)	CID: 3643	Anxiety disorder
Enalapril maleate salt (Sigma-Aldrich, USA)	CID: 5,388,961	Anxiety disorder
Sulfasalazine (Sigma-Aldrich, USA)	CID: 5339	Anti-inflammatory
Paracetamol (Atabay Kimya Sanayi ve Ticaret A.Ş, Türkiye)	SID: 178,101,923	Analgesic
Metoprolol tartrate salt (Sigma-Aldrich, USA)	CID: 441,308	Hypertension
Oxytetracycline hydrochloride (Dafeng Huashu Pharmaceutical Co. Ltd., China)	CID: 54,680,782	Antibiotic
Metronidazole (Aarti Drugs Ltd., India)	CID: 4173	Antibiotic
Clarithromycin (Zhejiang Guobang Pharmaceutical Co. Ltd., China)	CID: 84,029	Antibiotic
Amoxicillin trihydrate (DSM Sinochem Pharmaceuticals NL, The Netherlands)	CID: 62,883	Antibiotic
Piperacillin/tazobactam (Sterile India Pvt. Ltd., India)	CID: 9,918,881	Antibiotic
Naproxen sodium (Divis Laboratories Ltd., India)	CID: 23,681,059	Non-steroidal anti-inflammatory
Omeprazole (Chemo Laboratorios Liconsa, S.A., Spain)	CID: 4594	Proton pump inhibitor (stomach)

### Recombinant Production and Purification of *Ta*LDH

Briefly, recombinant *E. coli* BL21 (DE3) cells harboring the *Ta*LDH gene [38] were inoculated onto LB agar plates supplemented with 100 μg/ml ampicillin and incubated at 37 °C for 16 h. A single colony was transferred into liquid LB medium containing ampicillin and grown at 37 °C and 180 rpm. When the culture reached an OD₆₀₀ of 0.5–0.6, protein expression was induced with 0.5 mM IPTG, and the cells were incubated at 25 °C for 16 h before harvesting by centrifugation. After removing the supernatant, the pellets were resuspended in 50 mM Tris–KCl buffer, pH 7.5 [38]. Cell disruption was performed by sonication at 40% amplitude for a total of 2 min, consisting of six cycles of 10 s pulse followed by a 10 s interval. The lysate was subjected to centrifugation at 14,000 rpm for 20 min at 4 ℃. *Ta*LDH was subsequently purified using HisTALON® Gravity Columns [39]. The column was equilibrated with a buffer containing 50 mM NaH_2_PO_4_, 300 mM NaCl, and 10 mM imidazole at pH 8. The lysate was loaded onto the column, and the flow-through was collected. The column was then washed with wash buffer containing 50 mM NaH_2_PO_4_, 300 mM NaCl, 20 mM imidazole at pH 8, and the *Ta*LDH was eluted with elution buffer containing 50 mM NaH_2_PO_4_, 300 mM NaCl, 150 mM imidazole at pH 8. Finally, both elution and flow-through fractions were analyzed by sodium dodecyl sulfate–polyacrylamide gel (12%) electrophoresis (SDS-PAGE) [40].

Protein concentrations in the elution samples were determined using the Bradford assay [41]. In brief, bovine serum albumin (BSA) at different concentrations was used to generate the standard calibration curve. Reaction mixtures were prepared by combining 2 μL of BSA standard or elution sample with 200 μL of 1 × dye reagent and 798 μL of dH₂O, followed by brief vortex mixing. A blank solution containing 200 μL of 1 × dye reagent and 800 μL of dH₂O was prepared in parallel. The prepared samples were incubated in the dark at room temperature for 10 min, and after 200 μL of each reaction mixture was transferred to a 96-well microplate, absorbance was measured at 595 nm using a microplate spectrophotometer. A standard calibration curve equation (y = ax + b) was used for calculating the protein concentration of the elution samples.

### Inhibition test of APIs on *Ta*LDH

To evaluate the inhibitory potency of the ingredients, *Ta*LDH activity was measured in the presence of the inhibitor candidates dissolved in 99,7% (v/v) dimethyl sulfoxide (DMSO), corresponding to a final DMSO concentration of 0,997% (v/v) in a reaction. In a 96-well plate, a reaction mixture containing 0.15 mg/mL *Ta*LDH, 0.2 mM NADH, 0.1 mM of each API, and 50 mM Tris–KCl buffer (pH 7.5) was prepared and incubated for 10 min at room temperature [38]. After incubation, the enzymatic reaction was initiated by adding pyruvate to a final concentration of 0.01 M, and the reaction was monitored by measuring absorbance at 340 nm for 2 min using a BioTek Epoch UV Plate Reader. As a negative control, an equal amount of DMSO was added instead of the ingredients. All experiments were performed in triplicate.

### Structure Prediction of *Ta*LDH Using AlphaFold

The experimental determination of the three-dimensional crystal structure of *Ta*LDH remains unresolved; therefore, the structure model predicted by the AlphaFold server was selected for subsequent *in silico* studies. The PDB file of the three-dimensional structure of *Ta*LDH (UniProt accession number A0A3B0N5C5) predicted by AlphaFold Protein Structure Database [42] was downloaded and pLDDT (predicted Local Distance Difference Test) score ranges, which represent the confidence level of the predicted protein structure, where higher values indicate greater structural reliability, were evaluated. Score ranges are interpreted as > 90 as very high, 70–90 as medium–high, 50–70 as low-medium and < 50 as low confidence [43].

### Ligand and Protein Preparation

Three-dimensional structures of six APIs demonstrating over 60% inhibition of *Ta*LDH *in vitro,* were retrieved in SDF and PDB formats from the RSCB Protein Data Bank and DrugBank databases for *in silico* molecular docking studies. The molecules obtained in SDF format were transformed into PDB format with Open Babel (v3.1.1) software to render them compatible with AutoDock 4.2.6 [44]. Then, the ligands were imported into AutoDock Tools (ADT) 1.5.7, where their bonds were evaluated to identify rotatable bonds and enhance flexibility. Hydrogen atoms were incorporated into the APIs structures, Compute Gasteiger partial charges were computed [44], and each structure was transformed into PDBQT format and preserved following the specification of the molecules' flexible bonds.

The *Ta*LDH structure, whose three-dimensional conformation had been predicted by AlphaFold, was retrieved from the AlphaFold Protein Structure Database in PDB format. The removal of excess water molecules was unnecessary, as they were not present in the predicted *Ta*LDH PDB structure. Non-polar hydrogen atoms were incorporated into the structure to supplement absent hydrogen atoms, while the protein surface was charged by applying Kollman United Atom Charges [44]. The updated and protonated *Ta*LDH AlphaFold model was saved in PDBQT format.

### Grid Box Preparation and Molecular Docking

Potential ligand-binding sites of *Ta*LDH were analyzed using the DoGSiteScorer and Protein Plus servers [45]. AutoGrid 4.2.6 [44] was used to perform docking of APIs to *Ta*LDH, generating a grid box that encompassed the previously identified active site of *Ta*LDH. The grid box dimensions were established at 46 × 48 × 50 points along the x, y, and z axes, respectively. The grid spacing was chosen at 0.375 Å. These configurations were refined to facilitate the ligand's unrestricted movement within the binding pocket.

Docking studies of *Ta*LDH and six APIs (pantoprazole sodium, nateglinide, buspirone hydrochloride, paracetamol, piperacillin/tazobactam, and omeprazole), conducted using AutoDock 4.2.6 software, began with the selection of the Lamarckian Genetic Algorithm [46, 47] method for discovering binding poses. Docking parameters included a population size of 150, 2.5 × 10^6^ energy evaluations, and 27,000 generations. Ten independent docking runs were performed for each ligand to the *Ta*LDH, and the conformation with the lowest binding energy was selected as the most probable binding mode.

Binding energies were calculated using AutoDock’s semi-empirical free energy force field, which considers van der Waals interactions, hydrogen bonding, electrostatic forces, and desolvation effects [48].

## Results and Discussion

### Production and Purification Analyses of Recombinant *Ta*LDH

**Fig. 1:**
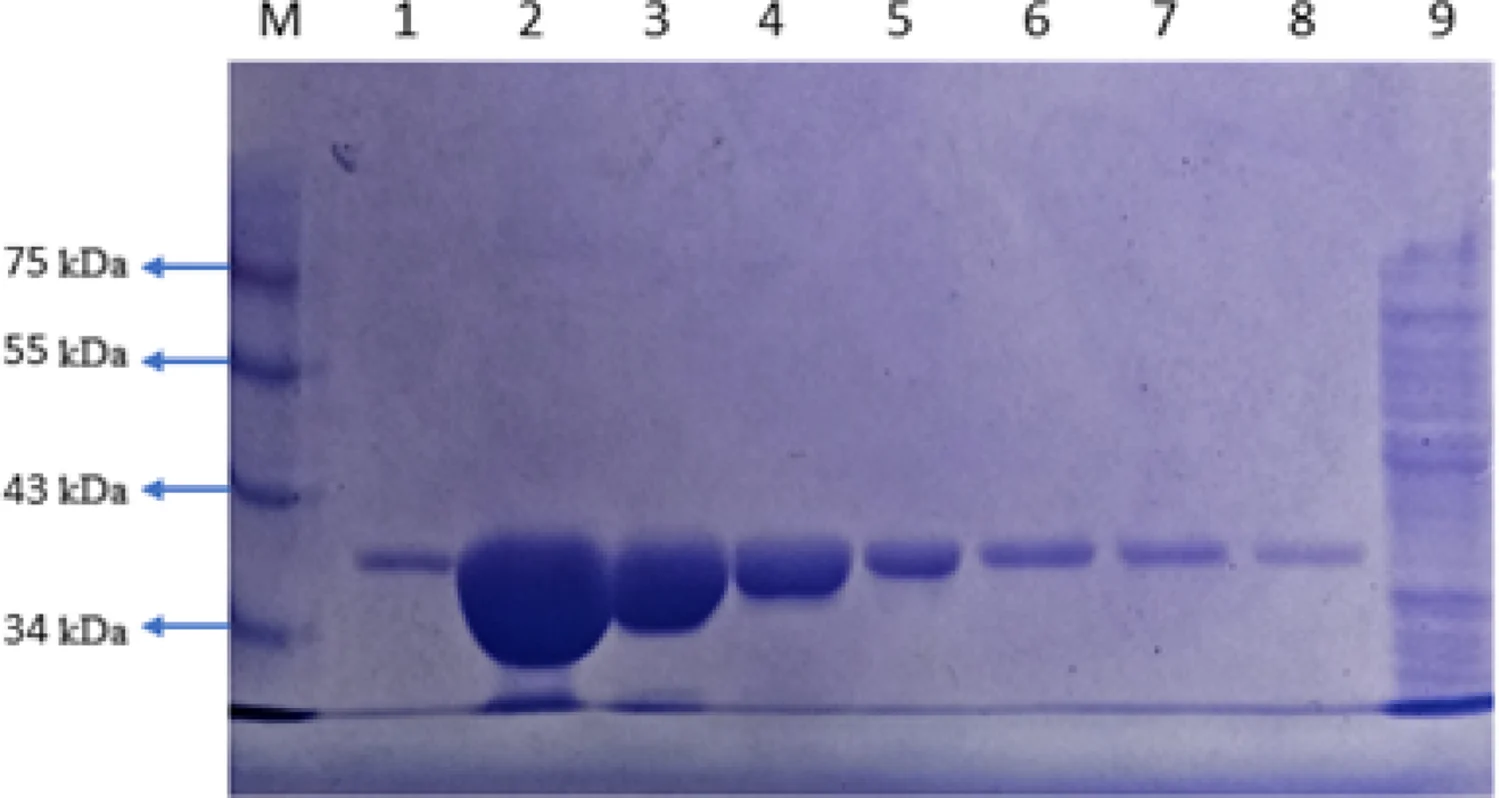
The SDS-PAGE image of elution and flowthrough fractions of *Ta*LDH; Line M: protein ladder; Lines 1, 2, 3, 4, 5, 6, 7, 8: elution fractions; Line 9: flowthrough

Recombinant *Ta*LDH was efficiently expressed in *E. coli* BL21 (DE3) cells, and the protein was purified using HisTALON® (cobalt (Co^2^⁺)-chelated TALON® resin) after inducing the cells with 0.5 mM IPTG at 25 °C for 16 h. The expression of approximately 35 kDa protein was confirmed by SDS-PAGE analysis (Fig. 1), consistent with the theoretical molecular weight of the protein that was calculated as 35.23 kDa using the Protein Molecular Weight tool in The Sequence Manipulation Suite server [49]. Elution fractions of *Ta*LDH exhibited intensely abundant protein band on the SDS-PAGE gel, with an estimated purity of about ~ 95%, as no extra protein band was observed (Fig. 1). The *Ta*LDH concentration was calculated as 3.755869 mg/mL (50 mL expression culture) using the Bradford assay [41]. Since high protein purity is crucial for ensuring the reliability of subsequent inhibition studies [50], the successful production of *Ta*LDH with high purity (over ~ 95%) and high yield provides a robust experimental foundation for *in vitro* biochemical inhibition screening assays.

### *In Vitro* Inhibition of *Ta*LDH by APIs

*In vitro* inhibition of *Ta*LDH was evaluated using each API at a final concentration of 100 µM. Triplicate measurements were performed for each API and compared with the DMSO negative control. Among the tested compounds, pantoprazole sodium, nateglinide, buspirone hydrochloride, paracetamol, piperacillin/tazobactam, and omeprazole exhibited more than 60% inhibition of *Ta*LDH. Their respective % inhibition values and standard errors are summarized in Table 2. The chemical structures of 6 APIs were obtained from the PubChem database [37].

**Table 2:** % inhibitory activities of APIs against *Ta*LDH at a concentration of 100 µM

Active pharmaceutıcal ıngredients of FDA-approved drugs	Structure	% ınhibiton value
Omeprazole		73.36 ± 5.894
Nateglinide		61.83 ± 1.257
Piperacillin/tazobactam		61.13 ± 9.947
Buspirone hydrochloride		66.81 ± 8.228
Paracetamol		65.16 ± 3.471
Pantoprazole sodium		60.76 ± 9.584

Percentage inhibition values are commonly used to assess the inhibitory potency of substances in enzymatic assays [51]. Beyond this, determination of % inhibition values enables quick identification and prioritization of the most effective inhibitors for further biochemical studies [52]. For instance, in high-throughput screening (HTS) studies, a defined threshold of percentage inhibition (i.e., 50%-100%) is a critical criterion for hit selection [53]. In a study by Ha et al. (2024), the dose-dependent inhibition of recombinant human LDHA by gossypol was investigated. It was reported that gossypol competitively inhibits LDHA by binding to NADH and, at approximately 10 μM, inhibits enzyme activity by 50%, demonstrating significant efficacy compared to the positive control, oxamate [54]. Furthermore, in an *in vitro* enzyme inhibition study to search for new inhibitors of L-lactate dehydrogenase from *Bacillus cereus* NRC1 (*Bc*-LDH), mangifrein (bioactive polyphenolic natural compound) was found to have an inhibition value of 58% at a concentration of 100 μM with a K_i_ value of 0.075 mM against recombinant *Bc*-LDH. In addition, *in silico* binding mode analysis highlights its potential as a novel inhibitor of LDH [55].

Six FDA-approved human therapeutic APIs analyzed in this study exhibited % inhibition values between 60.76 and 73.36 against *Ta*LDH, which may be considered promising; however, further *in vitro* and *in vivo* investigations are required to elucidate their potential use in the treatment of theileriosis and also to evaluate the safety, efficacy, and therapeutic potential of these APIs as alternative treatment options for theileriosis.

There are four categories of drug repurposing strategies: human-to-human, human-to-veterinary, veterinary-to-veterinary, and veterinary-to-human [36]. For instance, active ingredients used in humans are frequently used in veterinary treatments with similar, mild indications [56]. Repurposing approaches not only offer new indications for current drugs but also enhance accessibility for a greater number of patients and animals by lowering the costs [36].

In this study, some FDA-approved human therapeutic APIs, such as omeprazole, pantoprazole, nateglinide, piperacillin/tazobactam, buspirone, and paracetamol, are repurposed to evaluate their potential use in the treatment of *T. annulata* infections in cattle, presenting both therapeutic prospects and safety concerns.

Omeprazole, as a long-acting proton pump inhibitor (PPI), inhibits both basal and stimulated acid secretion. Due to this property, its use is authorized for the treatment of horses (*Equidae*) included in the food chain under EU Regulation 37/2010 [57]. PPIs such as omeprazole and pantoprazole effectively reduce abomasal acidity, increasing gastric pH and potentially supporting ulcer healing by providing mucosal protection in humans [58, 59]. Orally administering 4 mg/kg of omeprazole paste once daily has been shown to maintain abomasal pH above the critical level of 3 for 24 h in treated calves [58]. While PPIs such as omeprazole, lansoprazole, rabeprazole, and pantoprazole exhibit potent *in vitro* activity against protozoa, such as *Entamoeba histolytica*, *Giardia intestinalis*, and *Trichomonas vaginalis*, particularly pantoprazole, which demonstrates IC_50_ values in the nanomolar range [60], their efficacy against *T. annulata* warrants further investigation. Notably, incorporating these agents into bovine therapy requires careful clinical management to harmonize their anti-parasitic potential with physiological tolerance, as long-term use in calves has been noted to influence feed intake and metabolic profiles [61]. This study indicates that, in addition to omeprazole use in managing abomasal ulceration in cattle, it may serve as a supportive therapeutic agent for controlling theileriosis during the acute phase of infection. Given its 73.36% inhibition of *Ta*LDH, its short-term (intermittent) application could help reduce parasite burden while minimizing the risk of drug resistance associated with long-term exposure at sub-curative doses. Additionally, pantoprazole sodium, another proton pump inhibitor evaluated in this study, demonstrated 60.76% inhibition against *Ta*LDH. Given the antiparasitic potential suggested by Pérez-Villanueva et al. (2011) and the inhibition levels observed in this work, pantoprazole could also be proposed as a candidate for further evaluation as an alternative therapeutic agent for theileriosis. In this regard, pharmacokinetic and pharmacodynamic investigations are required to reveal the potential use of both drugs in the management of *Theileria* infections within the scope of drug repositioning.

Nateglinide stimulates insulin secretion by inhibiting K_ATP channels, resulting in a rapid hypoglycemic effect [62]. Hyperglycemia and hypocalcemia have been reported in goats infected with *Theileria* spp., and blood glucose levels rapidly return to normal with insulin treatment [63]. Given the observed 61.83% inhibition of nateglinide against *Ta*LDH in this study, the use of glucose-lowering agents such as nateglinide in cattle may contribute to treatment by limiting the parasite’s energy supply in cases where *T. annulata* disrupts glucose metabolism; however, the long-term metabolic effects require further investigations.

In a study of piperacillin/tazobactam use in ruminants, blood levels were monitored in horses administered piperacillin/tazobactam under different anesthesia protocols. It was determined that anesthesia significantly altered the blood level distribution, drug residence time, and excretion distribution [64]. Piperacillin/tazobactam, while microbiologically beneficial, may disrupt intestinal microbiota, increasing endotoxin levels, diarrhea risk, and potential food safety concerns due to milk excretion [65]. However, even when not used in combination with piperacillin/tazobactam, Singh's study on the treatment of cattle with pericarditis reported that combining tazobactam with cefquinone increased cefquinone's efficacy [66]. Further pharmacokinetic and toxicity profiles of piperacillin/tazobactam, which showed 66.81% inhibition against *Ta*LDH in this study, should be examined to evaluate its potential use in the fight against theileriosis.

In a study of ruminant horses, administration of the serotonin agonist buspirone for Equine Self-Mutilation Syndrome resulted in a significant reduction in self-mutilating behaviors, indicating that this neurotransmitter system may play a role in controlling the syndrome [67]. Buspirone, acting as a 5-HT1A receptor agonist, has been shown to inhibit catecholamine secretion in bovine adrenal cells [68], and its pharmacokinetic behavior in cattle, including serum binding, hepatic metabolism, and excretion, requires further characterization to ensure therapeutic safety [69–71].

Lastly, clinical symptoms in cattle with theileriosis include high fever and elevated body temperature (104º-106ºF) [72]. Paracetamol, commonly combined with meloxicam for fever control in bovine theileriosis [73], may aid in symptomatic relief but demands cautious dosing to prevent hepatotoxicity. Since paracetamol is already used to control fever in theileriosis, its dual use is suggested for drug repositioning, given that it inhibits *Ta*LDH by 65.16% according to this study.

Furthermore, all these findings suggest that more potent anti-theilerial agents could be developed through the targeted derivatization of each identified APIs exhibiting over 60% inhibition potential, followed by systematic lead optimization studies. Consequently, while these agents offer potential supportive or indirect therapeutic benefits, their pharmacokinetics, pharmacodynamic, and toxicological profiles in ruminants warrant comprehensive evaluation before clinical application.

### Molecular Docking of APIs to *Ta*LDH

Modern drug discovery is an integrated process with the sequential use of *in silico* and *in vitro* methodologies. A common approach involves first identifying promising compounds through virtual screening and then confirming their inhibitory potency *in vitro* [74]. Another approach involves initially identifying active chemicals using *in vitro* biochemical inhibition experiments, followed by elucidating their binding mechanisms by *in silico* analysis [75]. In this study, following assessment of the *in vitro* inhibition capabilities of six APIs against *Ta*LDH (pantoprazole sodium, nateglinide, buspirone hydrochloride, paracetamol, piperacillin/tazobactam, and omeprazole), molecular docking was conducted to determine the *in silico* binding poses of the APIs that demonstrated inhibition rates exceeding 60%. The reliability score of the *Ta*LDH structure predicted by AlphaFold, used in docking studies, has an average pLDDT [76] score of 94.38, with a pLDDT distribution including 88.8% very high, 6.2% high, 3.7% low, and 1.2% very low.

AutoDock was used to analyze DLG and receptor files and to assess binding energy, inhibition constant, and ligand efficiency. Structures exhibiting the most favorable binding energy were depicted as the optimal binding positions, consistent with *in vitro* findings. The docking analysis revealed the lowest energy conformations for each API as follows: pantoprazole sodium at − 5.14 kcal/mol, nateglinide at − 5.83 kcal/mol, buspirone hydrochloride at − 4.43 kcal/mol, paracetamol at − 4.2 kcal/mol, piperacillin/tazobactam at − 4.73 kcal/mol, and omeprazole at − 6.36 kcal/mol (Table 3). Furthermore, the ligand efficiency (kcal/mol/atom) and the inhibitor constant for each pose were computed using AutoDock. Based on the interactions of the best docking poses with adjacent residues, it was concluded that Pantoprazole sodium was unable to create a one-to-one hydrogen bond with surrounding residues; nateglinide formed a hydrogen bond with ARG161 (1.75 Å), no hydrogen bond interactions were observed for buspirone hydrochloride, paracetamol formed hydrogen bonds with ARG161 (2.057 Å), ASN130 (2.009 Å) and SER240 (1.918 Å), while piperacillin/tazobactam formed hydrogen bonds with ASN130 (2.019 Å) and HIS185 (1.851 Å), omeprazole also formed hydrogen bonds with ASN130 (1.669 Å) and HIS185 (1.976 Å).

**Table 3:** Binding energy (kcal/mol) and RMSD (Å) values obtained using AutoDock 4.2

APIs demonstrating over 60% inhibition	Binding energy/∆G (kcal/mol)	RMSD (Å)	Interactions with surrounding residues
Pantoprazol sodium	− 5.14	10.4	
Nateglinide	− 5.83	8.9	
Buspirone hydrochloride	− 4.43	10.38	
Paracetamol	− 4.2	8.9	
Piperacillin/tazobactam	− 4.73	4.74	
Omeprazole	− 6.36	9.21	

In the LDH active site, specific residues regulate substrate binding, transition state stabilization, and cofactor positioning. ARG171 mediates substrate binding, while HIS195 acts as the proton donor and is activated by ARG109 to stabilize the transition state [77]. ASP168 contributes to pKa modulation of HIS195, and VAL138 ensures proper cofactor orientation. ILE250 maintains the hydrophobic environment of the nicotinamide ring, whereas GLN102 and THR246 facilitate substrate release [77, 78]. These specific residues were also identified as key hydrogen-bonding interaction points with the substrate, suggesting a shared binding mechanism. This finding highlights a potential competitive or synergistic interaction at the active site. In the docking study of the substrate pyruvate with wild-type *Ta*LDH (wt*Ta*LDH), it was determined that pyruvate and wild-type wt*Ta*LDH formed several hydrogen bonds with the important residues ARG98 (109), ARG161 (171), and HIS185 (195) located in the substrate binding region [79]. In parallel with these findings, the APIs were found to form hydrogen bonds with HIS185 and ARG161, which are substrate-binding residues in the active site of the AlphaFold-predicted *Ta*LDH, according to docking results from this study.

Based on the binding energy and RMSD data in Table 3, the APIs exhibit interactions in the binding region; however, dynamic variables may result in different orientations compared to the crystal structure. The non-zero refRMS of the selected ligands at the lowest-energy position may be due to this position being considered to belong to a different cluster than the reference position. This generally indicates that the ligand has found different binding modes in quite different regions [80]. Although the high RMSD does not fully overlap with the reference position, interaction analysis indicates that the compounds make significant contacts with critical residues in the binding pocket, and these interactions may contribute to binding stability. When the *in vitro* *and* *in silico* findings were evaluated integratively, omeprazole exhibited both the highest percentage of inhibition and the lowest binding energy, demonstrating consistency between experimental and computational results.

## Conclusion

Successful drug discovery refers to a process that takes 10–15 years, with high failure rates and high costs; this process is particularly challenging for discovering anti-parasitic drugs [81]. Drug repurposing is a process that can be completed in 3–7 years, is approximately 85% more cost-effective than new drug development, and has a higher success rate [36]. Moreover, the safety, pharmacokinetic, and pharmacodynamic characteristics of these medications are pre-evaluated and confirmed in clinical environments, offering a benefit for repurposing [36]. In this study, APIs exhibiting 60% or higher inhibition, particularly omeprazole, should undergo advanced biochemical analyses to elucidate their inhibition kinetics. This preliminary *in vitro* enzymatic inhibition study suggests omeprazole as a potential therapeutic agent for theileriosis. Furthermore, these API’s may serve as promising leads for the future development of more potent anti-theilerial agents through targeted derivatization and lead optimization, after assessing anti-parasitic effect of these APIs of FDA-approved human therapeutics on *T. annulata* culture followed by evaluation of their host LDH activity and safety, and finally conducting comprehensive pharmacokinetic and pharmacodynamic analyses. Consistent with the One Health approach, evaluating human-approved active pharmaceutical ingredients as veterinary therapeutic options against parasitic diseases may offer a sustainable, time and cost-efficient strategy.

## Data Availability

The data generated and analyzed in this study is available from the corresponding author upon reasonable request.
